# Evaluation of robot-assisted surgery simulation skills after three days of intensive exposure – experience from intensive hands-on training courses

**DOI:** 10.3389/fsurg.2025.1564027

**Published:** 2025-06-26

**Authors:** Arif Özkan, Nikolaos Liakos, Rudolf Moritz, Martin Janssen, Özlem Kayaci-Güner, Markus Grabbert, Johannes Bründl, Burkhard Ubrig, Stefan Siemer, Christian Gratzke, Christian Wagner

**Affiliations:** ^1^ Department of Urology, Medical Faculty and Medical Centre of the University of Freiburg, Freiburg, Germany; ^2^ Department of Urology, Marien Hospital, Herne, Germany; ^3^ Department of Urology, University Münster, Münster, Germany; ^4^ European Robotic Institute Gronau, Gronau, Germany; ^5^ Department of Urology, Caritas Hospital St.Josef, Regensburg, Germany; ^6^ Department of Urology, Augusta Kranken-Anstalt Bochum, Bochum, Germany; ^7^ Department of Urology and Paediatric Urology, Saarland University, Homburg, Germany; ^8^ Department of Urology, Urological Oncology and Robot-assisted Surgery, St. Antonius Hospital Gronau, Gronau, Germany

**Keywords:** robotic-assisted surgery, simulation-based training, skills acquisition, Da Vinci Skills Trainer, intensive hands-on training, urological surgery, medical education, surgical education

## Abstract

**Introduction:**

Robot-assisted surgery represents a significant advancement in modern surgical techniques, offering the potential of unparalleled precision, flexibility, and control. Effective training of console surgeons is critical to harness these benefits. Simulation-based training, especially with virtual simulators like the Da Vinci Skills Trainer, plays a pivotal role in developing these essential skills. This study investigates the impact of intensive, short-term hands-on training courses on the simulation skills of robotic surgery trainees in Germany.

**Methods:**

We conducted a retrospective analysis of 52 participants from urological clinics with established robotic programs, who attended intensive training courses organized by the German Society of Robot-assisted Surgery (DGRU) and the Working Group (AK) Laparoscopy and Robot-Assisted Surgery of the German Society of Urology between 2018 and 2022 in a single training centre (IRCAD, Strasbourg, France), guided by experienced teachers. The training program included pre- and post-course evaluations using four specific exercises on the Virtual Reality Simulator (Da Vinci Skills Trainer): Ring Walk, Peg Board, Energy Dissection and Suture Sponge. Performance improvements were analyzed using paired *t*-tests. Statistically significant difference was considered as *p* < 0.05.

**Results:**

The results demonstrated significant improvements in participants’ skills across all evaluated exercises. The mean scores for the Ring Walk increased from 68.9 to 86.68 (*p* < 0.0001); Peg Board from 75.01 to 92.89 (*p* < 0.0001); Energy Dissection from 62.29 to 79.42 (*p* = 0.0377); and Suture Sponge from 61.41 to 79.21 (*p* < 0.0001). Notably, 78.84% of participants showed improvements in at least three of the four exercises, with an average score increase of 17%.

**Conclusion:**

Intensive simulation-based training was associated with improvements in robotic surgery simulation scores. These findings suggest that such training programs may help shorten the learning curve for novice surgeons and could contribute to improved readiness for clinical practice.

## 1 Introduction

In the landscape of modern surgery, robot-assisted techniques have emerged as a transformative advancement, offering enhanced precision, flexibility and control compared to traditional methods and the generally accepted benefits of minimally-invasive approaches. The training of console surgeons, who operate these sophisticated robotic systems, is crucial to realizing the full potential of robotic surgery. Simulation-based training has been recognized as an essential component in this educational paradigm, providing a safe and controlled environment for skill acquisition and refinement (1, 2).

Virtual simulators, such as the Da Vinci (DV) Skills Trainer (Intuitive Surgical, Sunnyvale, CA, USA), are integral to the early stages of console surgeon training. These simulators replicate the robotic surgical environment, allowing trainees to practice complex maneuvers without putting patients at risk. The exercises on these simulators typically focus on key skills, including endowrist manipulation, camera control, dissection, energy control and suturing (3). Previous studies have underscored the importance of simulation in improving surgical outcomes and reducing the learning curve for new surgeons (4, 5). The concurrent and predictive validity of robotic surgery simulators has been demonstrated, highlighting their effectiveness in skill improvement for surgical trainees with low baseline proficiency (6). Additionally, research confirmed the effectiveness of virtual reality (VR) simulator training in improving da Vinci performance, emphasizing that VR practice can lead to an early plateau in the learning curve for robotic procedures (7). Recent systematic reviews have provided comprehensive descriptions of training programs available for urological robotic surgery and endourology, assessing their validity and highlighting the fundamental elements for future training pathways (8).

Despite the established value of simulation training, there remains a need to evaluate the effectiveness of intensive, short-term exposure in hands-on training courses. Intensive training could potentially accelerate skill acquisition and optimization, providing a robust foundation for subsequent clinical practice. However, the impact of such training on the skill levels of participants has not been extensively studied in the context of robotic surgery in Germany.

This study aims to address this gap by evaluating the outcomes of participants in intensive hands-on training courses conducted by the German Society for Robot-assisted Surgery (DGRU) and the Working Group (AK) Laparoscopy and Robot-Assisted Surgery of the German Society of Urology (DGU). Specifically, the study assesses changes in simulation skills over a three-day training period, providing insights into the efficacy of intensive exposure to simulation-based training. By systematically documenting and analyzing the performance of participants before and after the training, this study seeks to determine whether intensive simulation training can lead to significant improvements in essential robotic surgery skills.

## 2 Material and methods

### 2.1 Study design and participants

This study is a retrospective analysis of participants who attended intensive hands-on training courses conducted by the DGRU and the AK Laparoscopy and Robot-Assisted Surgery between 2018 and 2022 in a single study centre (IRCAD; Strasbourg, France). A total of 52 participants were included in the study. The demographic details of the participants is given in Table 1. All participants were from urological clinics with established robotic programs, but themselves inexperienced in console surgery.

**Table 1:** The demographic details of the participants.

Participants, *n*	52
Gender, % (*n*)	♀: 34:6% (18) - ♂: 65.4% (34)
Mean age, years ± SD (range)	37 ± 5.3 (27–53)
Previous console experience, % (*n*)	23,1% (12)
Board certification status % (*n*)	71,15% (37)

### 2.2 training course structure

The training course was structured over three days, focussing on intensive hands-on training in manual dexteritxy in robot-assisted techniques. Experienced trainers conducted the sessions, providing real-time individualized feedback and guidance. The course included:

1. Pre-Course Evaluation (Day 1): Each participant's baseline skills were assessed using four specific exercises on the DV Skills Trainer:
  • Ring Walk: Evaluating endowrist manipulation and camera control.
  • Peg Board: Assessing fourth arm control, camera control, and clutching.
  • Energy Dissection: Measuring dissection and energy control.
  • Suture Sponge: Testing needle control and needle driving.

The initial evaluation provided baseline scores for each exercise, rated on a scale of 0–100.

2. Intensive Training (Days 1–3): Participants underwent rigorous training in robot-assisted techniques. The training included, among other various exercises:
  • Endowrist Manipulation: Enhancing dexterity and precision in controlling robotic instruments.
  • Control of the Fourth Arm: Developing coordination for using the additional robotic arm effectively.
  • Dissection and Energy Control: Practicing safe and efficient tissue manipulation and use of energy devices.
  • Needle Guidance and Suturing: Refining skills in needle handling and driving for suturing tasks.

Tasks were executed progressively—first virtually, afterwards in a dry lab setting—with inamite silicone and animal models, and subsequently in a wet lab setting, imitating real-case surgical steps and procedures.

The training sessions were designed to simulate real surgical scenarios, providing comprehensive learning experience. These sessions included dry lab tasks, wet lab tasks using the chicken gizzard for the urethrovesical anastomosis, the pyeloplasty training as well as the living swine model for radical prostatectomy and partial nephrectomy. The first two days comprised 16 h of extensive hands-on training altogether.

3. Post-Course Evaluation (Day 3): On the final day, participants repeated the same four exercises on the DV Skills Trainer to assess their progress. The post-course evaluation scores were documented for comparison with the baseline scores.

### 2.2 Data collection and analysis

The performance data for each participant were systematically documented. The key performance metrics included:

• Mean Scores Before and After Training: For each exercise.
• Range and Interquartile Range (IQR): To understand the distribution of scores.
• Average Improvement: Calculated as the difference between post-course and pre-course scores.

The data were analyzed using paired *t*-tests to compare the pre- and post-course scores. Prior to conducting the *t*-tests, the Kolmogorov–Smirnov test was performed to assess the normality of the data. A *p*-value of <0.05 was considered statistically significant. The paired *t*-tests were used to determine the statistical significance of the observed improvements in scores, with significant results indicating effective skills acquisition during the course.

The study was conducted in accordance with the ethical standards of the institutional and national research committees and with the Helsinki declaration and its later amendments.

## 3 Results

The study evaluated the outcomes of 52 participants who underwent 4 annual intensive hands-on training courses in robotic surgery between 2018 and 2022, with the exception of 2020 due to the Covid19 pandemic. The demographic profile of the participants revealed a diverse group, with 34.6% being female and 65.4% male. The age of the participants ranged from 27 to 53 years, with a mean age of 37 ± 5.3 years. Notably, 23.1% of the participants had previous some console experience, and 71.15% were already board-certified urology specialists (Table 1). All participants were affiliated with urological clinics that had established (or were to establish) robotic programs.

The training course's effectiveness was measured by comparing participants’ performance on the DV Skills Trainer exercises before and after the three-day intensive training. The exercises evaluated were “Ring Walk,” “Peg Board,” “Energy Dissection,” and “Suture Sponge,” focusing on various essential robotic surgery skills.

### 3.1 Ring walk

**Figure 1:**
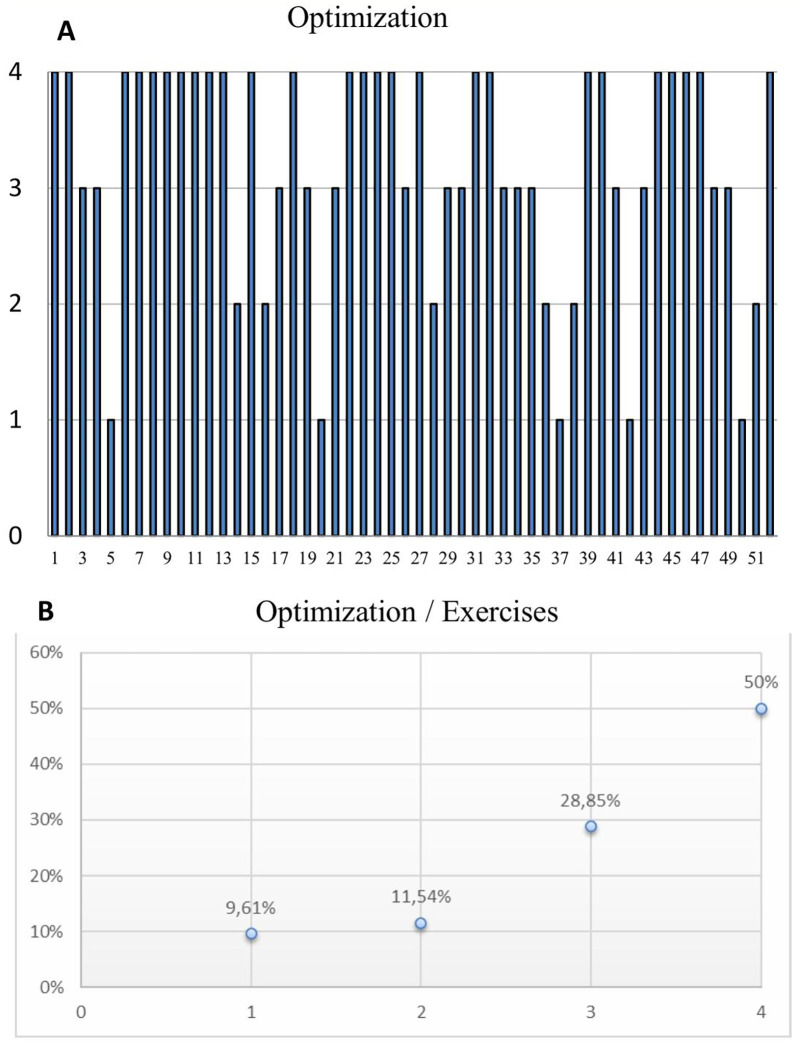
Composite figure includes box plots and bar graphs representing pre- and post-training performance distributions and individual optimizations for four different tasks (**(A)** ring walk, **(B)** Peg board, **(C)** energy dissection, **(D)** suture sponge). Each pair of box plots shows the score distributions before and after training, while the bar graphs illustrate individual performance changes, highlighting the training impact on each participant.

For the “Ring Walk” exercise, participants showed a significant improvement in their performance. The mean score before training was 68.90, which increased to 86.68 after the training. This represents an average improvement of 17.77 points. The range of scores before training was 0–99, with an interquartile range (IQR) of 40.55, while the range after training was 38–100, with an IQR of 19.75. The improvement was statistically significant, with a *p* < 0.0001 (Table 2, Figure 1).

**Table 2:** Exercise performance changes before/after and optimizations.

Exercises	Before		After		Optimization			*p*
	Mean (SD)	Median (IQR)	Mean (SD)	Median (IQR)	Range,	Average	Rate, % (*n*)	
					(*Δ* min) - (*Δ* max) (IQR)	(mean after - mean before)		
1. Ring Walk	68.90 (23.79)	74.30 (40.55)	86.68 (15.10)	89.25 (19.75)	(−6.50) - (+54.00) (30.55)	+17.77	88.46% (46)	<0.0001^t^
2. Peg Board	75.01 (27.54)	88.60 (35.10)	92.89 (8.01)	94.25 (5.17)	(−15.10) - (+93.00) (34.95)	+17.88	73.08% (38)	<0.0001^t^
3. Energy Dissection	62.29 (24.35)	66.30 (30.23)	79.42 (16.82)	80.95 (17.85)	(−33.90) - (+80.90) (25.95)	+17.13	82.69% (43)	0.0377^t^
4. Suture Sponge	61.41 (28.11)	67.70 (28.25)	79.21 (19.57)	83.00 (20.18)	(−89.00) - (+95.00) (24.98)	+17.8	86.54% (45)	<0.0001^t^

^t^ : Paired *t*-test; Δ min: minimum difference; Δ max: maximum difference.

### 3.2 Peg board

In the “Peg Board” exercise, the mean score before training was 75.01, which increased to 92.89 post-training, indicating an average improvement of 17.88 points. The score range before training was 0–100 (IQR: 35.10), and post-training it was 50.2–100 (IQR: 5.17). This significant improvement, with a *p* < 0.0001 (Table 2, Figure 1).

### 3.3 Energy dissection

For the “Energy Dissection” exercise, participants' mean score increased from 62.29 before training to 79.42 after training, reflecting an average improvement of 17.13 points. The pre-training score range was 0–99 (IQR: 30.23), and post-training it was 26.6–100 (IQR: 17.85). The improvement was statistically significant, with a *p* = 0.0377 (Table 2, Figure 1).

### 3.4 Suture sponge

In the “Suture Sponge” exercise, the mean score before training was 61.41, which increased to 79.21 after the training, showing an average improvement of 17.8 points. The score range before training was 0–100 (IQR: 28.25), and after training, it was 0–100 (IQR: 20.18). The improvement was statistically significant, with a *p* < 0.0001 (Table 2, Figure 1).

### 3.5 Overall improvement

**Figure 2:**
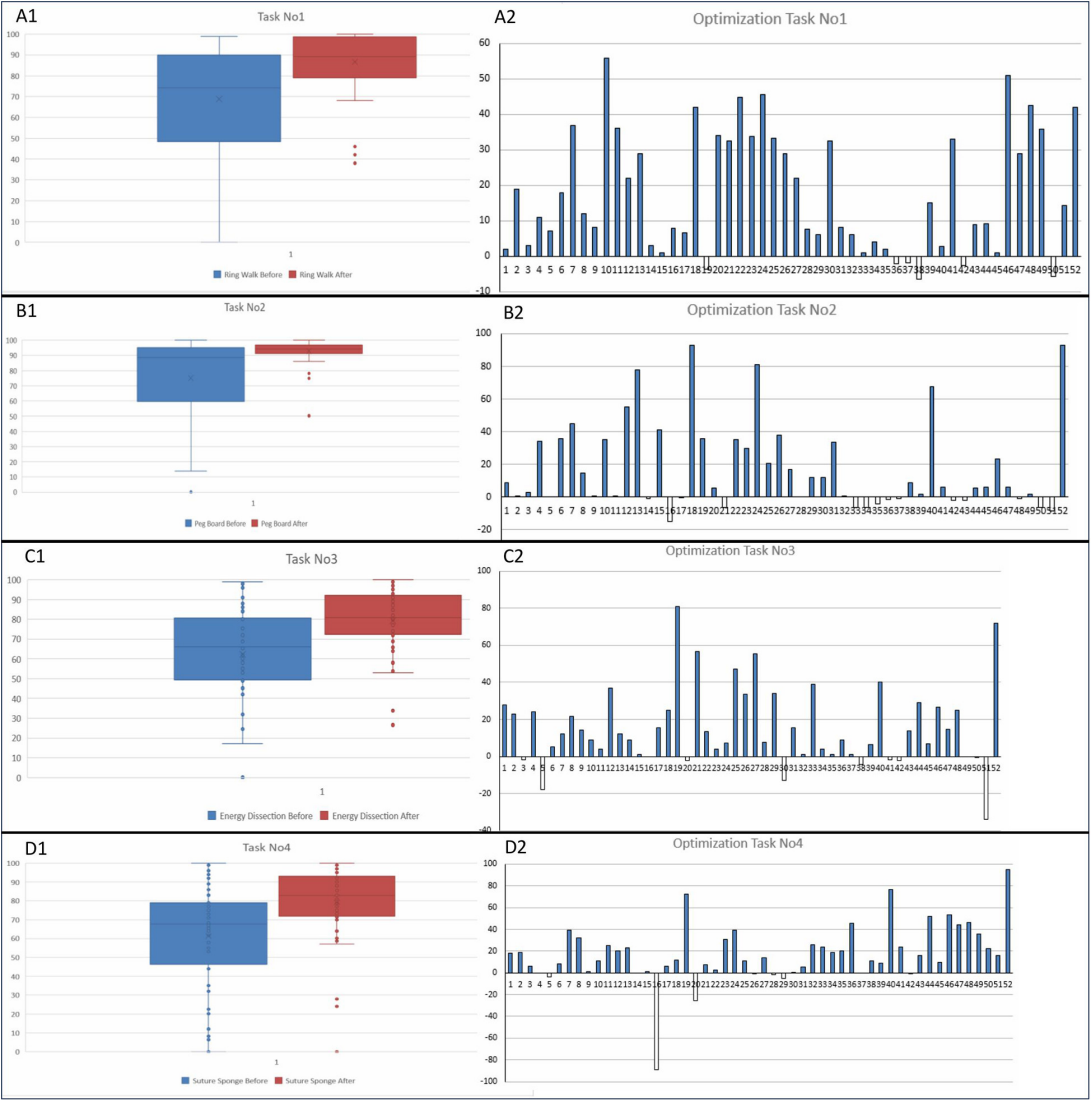
**(A)** Chart depicts the number of exercises in which each participant showed improvement after the three-day intensive training. Each bar represents a participant, with the height of the bar indicating the number of exercises (out of four) where they demonstrated better scores post-training. **(B)** Distribution of participants based on the number of exercises they improved in. It illustrates the percentage of participants who showed improvements in zero, one, two, three, or four exercises.

Overall, 78.84% of participants showed score improvements in at least three of the four exercises, with the average improvement across all exercises being 17%. Notably, half of the participants (*n* = 26) demonstrated enhancement across all four assessed exercises. Additionally, 28.85% (*n* = 15) improved in three exercises, while 11.54% (*n* = 6) and 9.61% (*n* = 5) showed improvements in two and one exercise, respectively. Importantly, none of the participants exhibited a lack of improvement in any area (Figure 2).

## 4 Discussion

This study demonstrates the significant impact of intensive, short-term simulation-based training on enhancing the skills necessary for robotic-assisted surgery. The marked improvements observed across all evaluated exercises underscore the effectiveness of such training programs.

Our results align with previous research highlighting the efficacy of simulation-based training in surgical education. Some studies reported substantial improvements in technical skills following simulation training, particularly in laparoscopic and robotic surgery (9, 10). Our study corroborates these findings, showing significant gains in endowrist manipulation, camera control, fourth arm coordination, dissection, and suturing skills. Specifically, the substantial improvements in the “Ring Walk” (mean score increase of 17.77 points, *p* < 0.0001) and “Peg Board” exercises (mean score increase of 17.88 points, *p* < 0.0001) confirm the critical role of focused training in enhancing these fundamental skills (9, 11).

The concurrent and predictive validity of the DV skills simulator has been evaluated, showing that simulator training significantly improved the skills of surgical trainees with low baseline proficiency, and simulation models have been shown to be beneficial not only before surgery but also as the first step in training, preceding animal models, dry lab exercises, and other training methods (6). However, their study involved a 10-week training period on the simulator. In contrast to this, our study examines the effects of an intensive short-term (three-day) simulation training. Our findings demonstrate that intensive short-term training can also lead to significant skills improvements, thereby contributing to the existing literature by showing the efficacy of shorter training programs. Various training models have been identified, and proficiency-based progression (PBP) curricula have been shown to have superior outcomes compared to traditional training methods (8).This supports the notion that structured and intensive training programs, even over a short duration, can effectively enhance surgical skills and should be integrated into surgical education frameworks. In our cohort, nearly 80% of participants demonstrated improvements in at least three of the four assessed simulator exercises. These findings suggest that intensive, hands-on training may be associated with short-term improvements in robotic surgery skills across multiple domains. While these results are promising, further investigation is warranted to determine whether such improvements translate into long-term clinical competence. Moreover, variation in improvement patterns among participants may indicate areas where the training curriculum could be further refined to maximize skill acquisition for all trainees.

While our study demonstrates immediate skill enhancement, the long-term retention of these skills remains an area for further investigation. Future studies should aim to evaluate the sustainability of skill improvements over extended periods and their translation into clinical outcomes. Longitudinal studies are needed to assess whether the gains in simulation scores translate to reduced operative times, fewer complications, and better patient outcomes in real-world settings. Proficiency-based training can enhance both technical skills and surgical performance, suggesting a potential for similar long-term benefits in robotic surgery training (12, 13).

The findings of this study may have significant implications for the design and implementation of surgical training programs worldwide. The success of our intensive training course supports its integration into broader surgical curricula, potentially accelerating the learning curve for new console surgeons. Institutions could adopt similar models, incorporating high-intensity, hands-on simulation training to enhance surgical competencies across various specialties. The effectiveness of virtual reality simulators in improving surgical skills and reducing learning curves have been highlighted in previous studies, which our findings further validate (12, 14).

Our study has several limitations, including a relatively small sample size and its single-institution design, which may affect the generalizability of the results. Additionally, the focus on short-term skill improvement without assessing long-term retention or practical application in clinical settings poses another limitation. A notable limitation of this study is the absence of a control group. Without a control cohort, it is not possible to definitively attribute the observed improvements to the training course itself. The score increases could partially be influenced by a test-retest effect, as participants may have performed better simply due to increased familiarity with the simulator tasks upon repetition. Future studies should incorporate randomized controlled designs to better assess the specific impact of intensive training interventions on skill acquisition. Future research should address these gaps by conducting multi-center studies with larger cohorts and longer follow-up periods. Exploring advanced metrics, such as motion analysis and haptic feedback, could provide deeper insights into the effectiveness of simulation training (4, 14). Additionally, collecting and analyzing qualitative feedback from participants may help refine training modules and increase their educational impact.

In light of our findings, it is worth noting that the effectiveness of robotic skills training appears to be more influenced by training methodology than by surgical background. A recent randomized controlled trial comparing PBP and traditional training across specialties (urology, gynecology, and general surgery) confirmed that trainees benefitted similarly from structured, metric-based feedback, regardless of their discipline (15). This reinforces the idea that fundamental robotic skills are transferable and that standardization of training methods could help address disparities in surgical education. However, despite the availability of validated curricula such as the ERUS program, access to comprehensive robotic training—including VR simulation, dry and wet labs, and modular console training—remains uneven worldwide due to logistical and financial barriers (16). These limitations raise important questions about equity in surgical education and underline the need for scalable training models. While our course demonstrated strong outcomes in a short timeframe, integration of PBP principles—especially its focus on error identification and mastery before progression—may further enhance the educational value of such intensive training interventions (17).

Our training program included several innovations, such as real-time performance feedback and scenario-based simulations that mimic actual surgical conditions. These features are consistent with the studies emphasized the importance of realistic training environments in improving surgical skills (11, 18). Future developments could further enhance these technologies, incorporating artificial intelligence to tailor training to individual learning curves and improving the overall effectiveness of simulation-based education.

Participant evaluation feedback was overwhelmingly positive, with many noting increased confidence and reduced anxiety in performing various steps during robotic procedures. This aligns with the study demonstrated that simulation-based training enhances both technical skills and psychological preparedness (13). Qualitative insights suggest that personalized feedback and scenario-based training were particularly beneficial, highlighting the need for continued refinement of training curricula to address diverse learning needs.

Finally, the cost-effectiveness of intensive simulation training vs. traditional methods warrants consideration. While our study did not perform a detailed cost-benefit analysis, existing literature indicates that simulation training can reduce costs associated with surgical errors and complications (1). By enhancing skill acquisition and reducing the learning curve, such training could potentially lead to significant economic savings and improved patient outcomes, reinforcing the value of investment in advanced training technologies.

## 5 Conclusion

This study suggests that intensive, hands-on simulation training may be effective in improving robotic surgery skills in the short term. While the results are promising, further controlled studies are needed to assess the long-term impact and clinical relevance of such training programs. These findings may support the development of structured training pathways to help prepare surgeons for the demands of modern robotic-assisted procedures.

## Data Availability

The original contributions presented in the study are included in the article/Supplementary Material, further inquiries can be directed to the corresponding author.
